# Neuroimaging of Anxiety in Parkinson's Disease: A Systematic Review

**DOI:** 10.1002/mds.28404

**Published:** 2020-12-02

**Authors:** Guillaume Carey, Meltem Görmezoğlu, Joost J.A. de Jong, Paul A.M. Hofman, Walter H. Backes, Kathy Dujardin, Albert F.G. Leentjens

**Affiliations:** ^1^ School for Mental Health and Neurosciences (MHeNS) Maastricht University Maastricht the Netherlands; ^2^ Université de Lille, Inserm, CHU Lille, Lille Neurosciences and Cognition Lille France; ^3^ Department of Psychiatry Maastricht University Medical Center Maastricht the Netherlands; ^4^ Department of Psychiatry, Ondokuz Mayis University Hospital Ondokuz Mayıs University Samsun Turkey; ^5^ Department of Radiology and Nuclear Medicine Maastricht University Medical Center Maastricht the Netherlands

**Keywords:** Parkinson's disease, anxiety, imaging, fear circuit, limbic circuit

## Abstract

**Background:**

The aim of this systematic review was (1) to identify the brain regions involved in anxiety in Parkinson's disease (PD) based on neuroimaging studies and (2) to interpret the findings against the background of dysfunction of the fear circuit and limbic cortico‐striato‐thalamocortical circuit.

**Methods:**

Studies assessing anxiety symptoms in PD patients and studies using magnetic resonance imaging, positron emission tomography, or single‐photon emission computed tomography were included.

**Results:**

The severity of anxiety was associated with changes in the fear circuit and the cortico‐striato‐thalamocortical limbic circuit. In the fear circuit, a reduced gray‐matter volume of the amygdala and the anterior cingulate cortex (ACC); an increased functional connectivity (FC) between the amygdala and orbitofrontal cortex (OFC) and hippocampus and between the striatum and the medial prefrontal cortex (PFC), temporal cortex, and insula; and a reduced FC between the lateral PFC and the OFC, hippocampus, and amygdala were reported. In the cortico‐striato‐thalamocortical limbic circuit, a reduced FC between the striatum and ACC; a reduced dopaminergic and noradrenergic activity in striatum, thalamus, and locus coeruleus; and a reduced serotoninergic activity in the thalamus were reported.

**Conclusion:**

To conclude, anxiety is associated with structural and functional changes in both the hypothesized fear and the limbic cortico‐striato‐thalamocortical circuits. These circuits overlap and may well constitute parts of a more extensive pathway, of which different parts play different roles in anxiety. The neuropathology of PD may affect these circuits in different ways, explaining the high prevalence of anxiety in PD and also the associated cognitive, motor, and psychiatric symptoms. © 2020 The Authors. *Movement Disorders* published by Wiley Periodicals LLC on behalf of International Parkinson and Movement Disorder Society

Fear is a universal emotion that triggers a state of alertness in response to a real or perceived threat. It may lead to a psychological and physiological state called anxiety and become a pathological symptom when the manifestations of anxiety are deleterious for the daily life of the subject, such as when the response is exaggerated or prolonged or occurs after exposure to inadequate stimuli. Anxiety is among the most frequent non‐motor symptoms in PD. The prevalence of anxiety in PD is 31%, which is higher than that reported in community or other medically ill patients.^1^ Although anxiety is a frequent worsening factor of the disease and is associated with lower quality of life,^2^, ^3^, ^4^ the underlying mechanisms remain largely unknown.

The fear circuit and the limbic cortico‐striato‐thalamocortical circuits play a parallel role in fear and anxiety. The fear circuit involves the amygdala and the anterior cingulate cortex (ACC), the medial prefrontal cortex (mPFC), the insular cortex, the hippocampus, and the striatum.^5^, ^6^, ^7^ The limbic cortico‐striato‐thalamocortical circuit involves the PFC, the basal ganglia, and the thalamus.^8^ In PD patients, alteration of these circuits such as dopaminergic, noradrenergic, and serotoninergic neurodegeneration may explain the high prevalence of anxiety.^9^

Several studies have explored the neural correlates of anxiety in PD using anatomical (magnetic resonance imaging [MRI]) and functional (positron emission tomography [PET], single‐photon emission computed tomography [SPECT], and functional MRI [fMRI]) neuroimaging. Although some systematic reviews of neuroimaging studies focusing on non‐motor symptoms in PD have been performed,^9^, ^10^, ^11^ none focused specifically on anxiety.

The aim of this systematic review was (1) to identify the brain regions involved in anxiety in PD patients based on the results of neuroimaging studies and (2) to interpret the findings against the background of dysfunction of the fear and the limbic circuits.

## Patients and Methods

The protocol for this systematic review was registered in PROSPERO and followed the PRISMA guidelines^12^, ^13^ (PROSPERO‐ID CRD42020158980). A literature search in PubMed/Medline, PsychINFO, and the Cochrane Library was performed using these search terms: (Parkinson's disease OR Parkinson) AND (Anxiety) AND ([imaging] OR [MRI] OR [PET] OR [SPECT] OR [fMRI] OR [functional MRI]). The search was conducted across the entire time span until January 8, 2020, and resulted in 382 articles. Further information about data selection and inclusion criteria is provided in Supplementary Methods S1 in Appendix S1.

A quality assessment to assess the risk of bias in individual studies has been performed using 9 quality criteria, following the approach of Wolters et al.^14^ More information about data extraction and quality assessment is provided in Supplementary Methods S2 in Appendix S1. The data selection, quality assessment, and data extraction were performed by two authors independently (G.C. and M.G.), and discrepancies were discussed until a consensus was reached.

Imaging data were summarized in 3 tables: anatomical, functional, or metabolic differences. In each table, the localizations of these changes were identified according to their peak coordinates in Montreal Neurological Institute space. The main changes were considered relevant according to their frequency and reproducibility between all the studies. No statistical test was used for this systematic review. Relevant changes were reported on figures representing cortical or subcortical structures to summarize and to better visualize these changes.

## Results

### Research Results

Eighteen imaging studies met the inclusion criteria and were included in this systematic review. These consisted of 4 anatomical MRI studies,^15^, ^16^, ^17^, ^18^ 4 fMRI studies,^19^, ^20^, ^21^, ^22^ 8 neurotransmitter/transporter imaging studies^23^, ^24^, ^25^, ^26^, ^27^, ^28^, ^29^, ^30^ and 2 metabolic imaging studies.^31^, ^32^ No computed tomography study was found. The flow chart of the study selection procedure is provided in Supplementary Results S3 in Appendix S1. Taken together, the included studies comprised 1840 participants (1470 PD patients and 370 healthy controls [HCs]). Demographic characteristics are presented in Table 1. According to the quality assessment, 12 of 18 studies received a score of “good,”^15^, ^16^, ^17^, ^18^, ^19^, ^20^, ^22^, ^23^, ^26^, ^29^, ^30^, ^32^ and 6 received a score of “moderate.”^21^, ^24^, ^25^, ^27^, ^28^, ^31^ Further information about this quality assessment can be found in the Supplementary Methods S2 in Appendix S1.

**TABLE 1:** Demographic and clinical characteristics of the participants in the studies included in the systematic review

		Age	Gender	Education	Disease duration	LEDD				Cognition
Study	Size	(y)	(M/F)	(y)	(y)	(mg/day)	UPDRS‐III	Anxiety scale	Depression scale	(MMSE/MoCa*)
**Anatomical imaging studies**										
Oosterwijk et al^15^								BAI	BDI	
PD	115	63.9 (±11.0)	71/44	–	3.6 (±4.5)	164.5 (±290.2)	24.7 (±11.3)	11.7 (±8.3)	11.0 (±7.5)	28.4 (±1.5)
Ma et al^18^								HAMA	HAMD	
aPD	8	65.75 (±8.41)	2/6	11.88 ± 4.05	8.88 (±5.74)	593.16 (±293.77)	30.75 (±11.06)	17.63 (±3.11)	10.50 (±3.21)	28.00 (±3.34)
naPD	33	65.27 (±9.09)	17/16	13.52 ± 2.73	7.67 (±4.11)	401.28 (±246.05)	28.09 (±10.62)	6.45 (±3.17)	6.64 (±2.91)	28.42 (±1.35)
Vriend et al^16^								BAI	BDI	
PD	110	64.6 (±10.3)	66/44	–	3.3 (±3.6)	436.4 (±332.7)	24.9 (±10.4)	12.3 (±8.3)	10.2 (±7.1)	28.4 (±1.5)
Wee et al^17^								HADS‐A	GDS	
PD	73	65.19 (±7.99)	56/17	11.03 (±3.21)	4.85 (±3.10)	595.88 (±398.99)	18.42 (±8.20)	4.53 (±3.37)	2.81 (±2.82)	26.42 (±2.91)*
**Functional imaging studies**										
Zhang et al^22^								SAS	SDS	
PD	36	62.98 (±6.61)	30/6	–	6.73 (±4.21)	928.46 (±132.82)	20.86 (±10.81)	31.42 (±4.67)	32.53 (5.85)	28.94 (±1.19)
Wang et al^21^								HAMA	HAMD	
aPD	15	71.33 (±5.27)	10/5	12.13 (±2.72)	4.27 (±3.44)	454.03 (±262.34)	24.80 (±9.90)	15.00 (±3.21)	8.93 (±2.34)	27.53 (±2.03)
naPD	33	69.48 (±6.03)	24/9	11.48 (±3.95)	4.43 (±3.00)	441.48 (±291.27)	22.73 (±10.82)	6.61 (±3.34)	4.24 (±3.39)	28.09 (±1.70)
HC	19	66.21 (±3.51)	10/9	10.58 (±3.24)	NA	NA	NA	1.79 (±1.62)	1.47 (±0.90)	28.79 (±1.03)
Dan et al^19^								STAI	BDI	
PD	27	64.9 (±7.9)	15/12	13.5 (±2.7)	11.1 (±3.7)	1306.1 (±616.7)	14.4 (±7.1)	(S) 38.7 (±9.4) (T)41.8 (±8.9)	10 (±4.8)	26 (±2.2)*
Wang et al^20^								HAMA	HAMD	
aPD	18	71.74 (±5.16)	12/6	12.84 (±2.95)	3.76 (±3.23)	450.17 (±252.08)	24.05 (±8.92)	15.47 (±3.01)	9.26 (±2.64)	27.79 (±1.87)
naPD	45	66.17 (±8.11)	34/11	11.52 (±3.56)	3.94 (±2.87)	373.95 (±306.93)	21.52 (±10.59)	5.93 (±3.42)	3.83 (±3.19)	28.24 (±1.57)
HC	24	65.33 (±4.65)	10 /14	10.79 (±2.92)	NA	NA	NA	2.33 (±2.04)	1.54 (±2.06)	–
**Neurotransmitter/transporter imaging studies**										
Bayram et al^30^										
PD‐L	154	60.3 (±9.86)	88/66	15.4 (±3.07)	0.52 (±0.53)	–	21.6 (±8.38)			
PD‐R	213	62.3 (±9.59)	147/66	15.6 (±3.10)	0.55 (±0.54)	–	19.9 (±8.77)	Not shown	Not shown	Not shown
HC	113	60.8 (±12.2)	65/48	16.3 (±3.04)	NA	–	NA			
Joling et al^29^								BAI	BDI	
PD	127	64.91 (±10.98)	84/43	–	2.55 (±2.90)	161.77 (±274.78)	23.02 (±10.68)	11.50 (±8.32)	8.00 (±9.00)	Not shown
Picillo et al^28^								STAI	GDS	
PD	405	61.20 (±9.8)	264/140	15.56 (±2.98)	–	–	20.25 (±8.93)	65.35 (±18.47)	2.29 (±2.37)	–
HC	187	60.24 (±11.2)	121/66	16.12 (±2.9)	–	–	NA	57.03 (±14.33)	1.28 (±2.08)	–
Ceravolo et al^27^								HAMA	HAMD	
PD	44	68.1(±7.9)	–	–	13.7 (±11.7)	–	17.9 (±7.7)	3 (±3.6)	4.1 (±5.0)	26.9 (±1.5)
Erro et al^26^								HADS‐A	BDI	
aPD	9	58.7 (±9.4)	4/5	–	14.9 (±3.5)	–	15.5 (±5.7)	≥7	5.9 (±7.3)	28.2 ± 0.9
naPD	25	59.5 (±8.3)	18/7	–	16.2 (±3.1)	–	13.3 (±6.1)	<7	7.5 (±6.8)	27.4 (±2.2)
Moriyama et al^25^						–		BSPS		
sad_PD	12	50.5 (±11.3)	9/3	–	7.1 (±3.8)	–	34.7 (±16.1)	56.5 (±11.3)	–	–
nosad_PD	20	52.5 (±12.8)	15/5	–	9 (±6.2)		31.7 (±12.2)	25.7 (±14.2)	–	–
Weintraub et al^24^								STAI	POMSd	
PD	76	62.8 (±10.8)	57/19	15.1 (±2.9)	7.5 (±5.5)	–	–	(S)37.5 (±9.0) (T) 37.0 (±7.6)	6.0 (±7.4)	–
Remy et al^23^								STAI	BDI	
PD	20	58.15 (±8.1)	14/6	–	4 (±2.2)	501.9 (±415.6)	23.8 (±8.95)	41.3 (±12.65)	12.3 (±4.75)	–
**Metabolic imaging studies**										
Wang et al^31^								HAMA	HAMD	
aPD	13	68.31 (±5.71)	9/4	10.62 (±2.33)	3.85 (±2.72)	297.88 (±185.29)	21.31 (±10.04)	14.08 (±3.04)	5.46 (±3.26)	29.15 (±0.99)
naPD	15	64.13 (±8.95)	8/7	10.67 (±2.87)	2.44 (±2.65)	190.83 (±256.28)	15.60 (±9.24)	5.33 (±3.11)	5.13 (±2.90)	28.93 (±1.22)
HC	15	63.33 (±4.62)	8/7	10.00 (±3.16)	NA	NA	NA	1.87 (±1.85)	4.53 (±2.23)	29.00 (±0.85)
Huang et al^32^								BAI	BDI	
PD	26	66.5 (±1.4)	16/10	17.8 (±0.6)	5.5 (±0.7)	–	Not shown	12 (1–35)	8 (1–21)	29 (26–30)
HC	12	67.4 (±2.0)	7/5	17.2 (±1.1)	NA	NA	NA	2 (0–10)	3 (0–10)	30 (30–29)
Total										
PD	1470	63.40 (±5.26)	1114/356	13.55 (±2.43)	6.26 (±4.47)	510.13 (±363.23)	20.91 (±4.87)	NA	NA	NA
HC	370	63.89 (±2.93)	221/149	14.44 (±3.88)	NA	NA	NA	NA	NA	NA
Total	1840									

Abbreviations: aPD, PD with anxiety; BAI, Beck Anxiety Inventory; BDI, Beck Depression Inventory; BSPS, Brief Social Phobia Scale; GDS, Geriatric Depression Scale; HADS‐A, Hospital Anxiety and Depression Scale, Anxiety Subscale; HAMA, Hamilton Rating Scale for Anxiety; HAMD, Hamilton Depression Rating Scale; HC, healthy controls; LEDD, levodopa equivalent daily dosage; MMSE, Mini‐Mental State Examination; MoCA, Montreal Cognitive Assessment; NA, not applicable; naPD, PD without anxiety; PD, Parkinson's disease; POMSd, Profile of Mood State—Depression Scale; sad, social anxiety disorder; SAS, Self‐Rating Anxiety Scale; (S), state; SDS, Self‐Rating Depression Scale; STAI, Spielberg State‐Trait Anxiety Inventory; (T), trait; UPDRS, Unified Parkinson's Disease Rating Scale, *cognition assessed using MoCA.

### Anatomical MRI Studies

The 4 anatomical MRI studies together comprised 329 PD patients. None included HCs. All were based on 3‐T MRI T1‐weighted scans. Three studies used voxel‐based morphometry (VBM) to analyze gray‐matter volume, and 1 used structural covariance analyses to analyze structural connectivity. Two studies compared PD patients with and without anxiety (aPD and naPD), and 2 studies correlated the severity of anxiety to anatomical changes. The studies used three different scales for the assessment of anxiety: the Beck Anxiety Inventory (BAI),^33^ the Hamilton Rating Scale for Anxiety (HAMA),^34^ and the Hospital Anxiety and Depression Scale, Anxiety Subscale (HADS‐A).^35^

In studies using VBM, higher anxiety scores, as measured with the BAI, were associated with a reduced volume of the bilateral ACC, the left amygdala, the bilateral precuneus, and the bilateral cerebellar tonsils. There were negative correlations between the BAI and structural covariance of the left striatum and right caudate and between the left striatum and bilateral prefrontal cortex (PFC). The results are presented in Table 2.

**TABLE 2:** Anatomical imaging studies of PD‐related anxiety

Studies	Size	Anxiety scale	Outcome	Analyze software	Localization	MNI coordinates	Statistic values
						*x*/*y*/*z*	
Oosterwijk et al^15^					Negative correlation		*z* Scores
PD	115	BAI	Structural covariance	Multiple regression	l. DCN	−13/15/9	5.36
					r. caudate	12/18/14	5.33
				SPM	r. DCN	13/15/9	4.71
					r. vlPFC	51/30/−4	5.48
					l. DCP	−28/1/3	4.79
					r. caudate	10/16/14	
					l. NA	−9/9/−8	
					r. caudate	10/16/12	
					l. dlPFC	−48/20/40	
Ma et al^18^							*z* Scores
aPD	8	HAMA	GMV (VBM)	Comparisons (ANOVA)	r. tonsil/lobule VIII	34.5/−48/−43.5	2.92
naPD	33			SPM	l. tonsil	−40.5/−46.5/−43.5	2.76
Vriend et al^16^				Multiple regression			T‐values
PD	110	BAI	GMV (VBM)	FreeSurfer, SPM	l. amygdala	−24/0/−29	2.91
Wee et al^17^							*z* Scores
PD	73	HADS‐A	GMV (VBM)	Multiple regression	l. precuneus	−18/−63/36	3.69
					l. ACC	−8/23/28	3.70
				SPM	r. precuneus	12/−55/36	3.73
					r. ACC	8/30/15	3.36

Abbreviations: ANOVA, one‐way analysis of variance; aPD, PD patients with anxiety; BAI, Beck Anxiety Inventory; GMV, gray‐matter volume; HADS‐A, Hospital Anxiety and Depression Scale, Anxiety Subscale; HAMA, Hamilton Rating Scale for Anxiety; MNI, Montreal Neurological Institute; naPD, PD patients without anxiety; PD, Parkinson's disease; SPM, statistical parametric mapping; VBM, voxel‐based morphometry.

Region of interest: ACC, anterior cingulate cortex; DCN, dorsal caudate nucleus; DCP, dorsal‐caudate putamen; dlPFC, dorsolateral prefrontal cortex; IC, insular cortex; l, left; NA, accumbens nucleus; PCC, posterior cingulate cortex; preCG, precentral gyrus; r, right; SFG, superior frontal gyrus; vlPFC, ventrolateral prefrontal cortex.

### fMRI Studies

The 4 fMRI studies comprised 217 participants, of whom 174 were PD patients and 43 were HCs. In all studies, 3‐T resting‐state fMRI and T1‐weighted scans were performed. In all studies, voxel‐level seed‐based analysis was performed, and in 1 study, an additional region of interest–level analyses was performed. Functional connectivity strength between an identified seed and the whole brain was performed in three studies, whereas in 1 study the amplitude of low‐frequency fluctuations (ALFFs) in the whole brain was analyzed, corresponding to the functional activity. In 2 studies aPD, naPD, and HC were compared, whereas in 2 studies the severity of anxiety was correlated with functional changes. No study was found using diffusion tensor imaging (DTI). Three different anxiety rating scales were used: the HAMA, Self‐Rating Anxiety Scale,^36^ and the Spielberg State‐Trait Anxiety Inventory (STAI).^37^

In aPD patients, higher ALFFs were reported in the right cerebellum (regions IX and VIII) and the right orbitofrontal cortex (OFC) than naPD or HC. Increased anxiety was associated with a stronger functional connectivity (FC) between the amygdala and the OFC, parietal cortex (more specifically the superior parietal lobule, precuneus, and angular gyrus), and the medial temporal cortex. Moreover, there was stronger FC between the OFC and temporal cortex, between the striatum and temporal cortex, and between the striatum and the cingulate cortex. Increased anxiety severity was associated with a lower FC between the amygdala and the dorsolateral prefrontal cortex (dlPFC), between the striatum and the OFC, and between the OFC and dlPFC. The results are presented in Table 3.

**TABLE 3:** Functional imaging studies of PD‐related anxiety

Studies	Size	Anxiety scale	Outcome	Analyze software	Localization	MNI coordinates		Statistic values	
						*x*/*y*/*z*			
Zhang et al^22^ PD	36	SAS	Weighted degree and FC strength (BOLD signal)	Correlations (GLM) SPM, RESTplus	Anxiety			T‐values	
					FC l. amygdala.	−21/0/−12			
					l. AG	−54/−63/33		6.15	
					l. SPL	−36/−69/48		5.54	
					l. cuneus	−9/−87/6		5.25	
					r. IFG	42/36/9		−5.74	
					l. STG	−63/−33/12		−5.39	
Wang et al^21^ aPD naPD HC	15 33 19	HAMA	ALFF methods	Comparisons (ANCOVA) SPM rs‐fMRI data analyses toolkit	aPD ≥ naPD			*z* score	
					r. cereb.IX	9/−42/−51		4.07	
					r. cereb.VIII	18/−72/−42		4.40	
					r. OFC	33/51/9		4.44	
					aPD ≥ HC				
					r. cereb.VIII	21/−72/−42		4.24	
					r. OFC	27/48/3		4.11	
					r + l. medulla	6/−42/−51		4.24	
Dan et al^19^ PD	27	STAI	FC strength (BOLD signal)	Multiple regression Software "CONN" (Matlab)	Anxiety	Left	Right	T‐values	
					FC OFC	−5/37/−18	8/36/−18	Left	Right
					Amyg.	−23/−1/−17	27/1/−18	3.73	4.19
					Hipp.	−25/−21/−10	29/−20/−10	4.35	3.81
					ParaHipp.G	−21/−16/−21	25/−15/−20	5.38	7.36
					FC iMTG	–	57/−37/−1		
					OFC	–	8/36/−18	ns	3.94
					Amyg.	–	27/1/−18	ns	4.9
					Hipp.	–	29/−20/−10	ns	4.55
					ParaHipp.G	–	25/−15/−20	ns	3.95
					FC SMC	−39/−6/51	41/−8/52		
					OFC	−36/31/−12	18/48/−14	−5.02	−4.04
					FC dlPFC	−5/54/−7	8/52/−7		
					Amyg.	−23/−1/−17	27/1/−18	−4.26	−5.18
					TP	−40/15/−20	–	−4.38	ns
					OFC	−36/31/−12	–	−5.01	ns
Wang et al^20^ aPD naPD HC	18 45 24	HAMA	FC strength (BOLD signal)	Comparisons (ANCOVA) SPM	aPD ≥ naPD			*z* Values	
					FC l. putamen	−24/4/2			
					r. OFC	13/18/60		−3.130	
					FC r. putamen	28/5/2			
					l. OFC	−6/63/−3		−3.744	
					r. cereb.	51/−63/−48		−5.199	
					r. precuneus	0/−45/72		−3.981	
					r. IC	39/−9/−6		4.713	
					l. TP	−39/−3/−15		4.343	
					l. MOG	−42/−87/−3		3.162	
					l. caudate	−15/15/18		3.976	
					r. MCC	12/−6/33		3.208	
					aPD ≥ HC				
					FC l. putamen	−24/4/2			
					l. ACC	−12/36/3		−4.136	
					FC r. putamen	28/5/2			
					l. OFC	−6/39/−9		−3.490	
					r. paraCL	6/−24/75		3.590	
					l. paraCL	0/−30/63		3.755	

Abbreviations: ANCOVA, analysis of covariance; BOLD, blood‐oxygen‐level‐dependent; BR, binding rate; ALFF, amplitude of low‐frequency fluctuations; aPD, PD patients with anxiety; HAMA, Hamilton Rating Scale for Anxiety; HC, healthy controls; FC, functional connectivity; GLM, generalized linear model; ns, not significant; MNI, Montreal Neurological Institute; naPD, PD patients without anxiety; PD, Parkinson's disease; SAS, Self‐Rating Anxiety Scale; SPM, statistical parametric mapping; STAI, Spielberger State‐Trait Anxiety Inventory.

Region of interest: ACC, anterior cingulate cortex; AG, angular gyrus; amyg, amygdala; cereb, cerebellum; dlPFC, dorsolateral prefrontal cortex; hipp, hippocampus; IC, insular cortex; IFG, inferior frontal gyrus; iMTG, inferior middle temporal gyrus; l, left; MCC, middle cingulate cortex; MOG, middle occipital gyrus; OFC, orbitofrontal cortex; paraCL, paracentral lobule; r, right; SMC, sensorimotor cortex; SPL, superior parietal; STG, superior temporal gyrus; TP, temporal pole.

### Neurotransmitter/Neurotransporter Studies

The 8 neurotransmitter/neurotransporter imaging studies comprised 1292 participants, of whom 1105 were PD patients and 187 were HCs. In 6 studies, the dopamine transporter (DAT) binding rate (BR) in the striatum was analyzed using ^99m^Tc‐TRODAT‐1 SPECT (2 studies) or ^123^I‐FP‐CIT SPECT (4 studies). In 1 study, the DAT and noradrenaline transporter (NAT) BR were analyzed using ^11^C‐RTI‐32 PET. In another study, the DAT and serotonin transporter (SERT) BR were analyzed using ^123^I‐FP‐CIT SPECT. In 5 studies, aPD patients were compared with naPD patients or HCs. Eight studies correlated the severity of anxiety with changes in the BR. Five different anxiety scales were used: the STAI, the BAI, the HAMA, the Brief Social Phobia Scale),^38^ and the HADS‐A.

Increased anxiety in PD was associated with reduced DAT binding in the bilateral caudate, the left putamen, the bilateral thalamus, bilateral amygdala, and the left locus coeruleus. Increased anxiety was also associated with reduced NAT in the left caudate, the bilateral thalamus, the bilateral amygdala, and the left locus coeruleus, as well as with reduced SERT in the bilateral thalamus. Two studies focused specifically on social anxiety disorders.^25^, ^27^ Both reported that severity of social anxiety was associated with increased DAT binding in the striatum, bilaterally. The results are presented in Table 4.

**TABLE 4:** Metabolic imaging studies of PD‐related anxiety

Studies	Size	Anxiety scale	Imaging/outcome	Analyze software	Localization	MNI coordinates	Statistic values
						*x*/*y*/*z*	
**Neurotransmitter/transporter imaging (PET/SPECT) studies**							
Bayram et al^30^ PD‐L PD‐R HC	154 213 113	STAI	^123^I‐FP‐CIT SPECT Striatal DAT BR	Correlation: mixed model SAS	PD‐L STAI‐state	Not available in this study	r‐Values (*P*‐values)
					r. caudate		−0.11 (0.039)
					l. caudate		−0.19 (<0.001)
					l. putamen		−0.13 (0.006)
					STAI‐trait		
					r. caudate		−0.13 (0.029)
					l. caudate		−0.20 (<0.001)
					l. putamen		−0.14 (0.004)
					No correlation with PD‐R and HC		
Joling et al^29^ PD	127	BAI (affective subscale)	^123^I‐FP‐CIT SPECT Striatal DAT Extrastriatal SERT BR	Multiple regression SPM Software "FMRIB"	BAI_affective_ ROI level		β‐Values (*P*‐value)
					r. thalamus	–	−0.203 (0.019)
					VBM		T‐values
					l. thalamus	−14/−24/0	−4.11
Picillo et al^28^ PD HC	405 187	STAI	^123^I‐FP‐CIT SPECT DAT BR	Multiple regression Software "Hermes" and "Pmod"	STAI‐trait subscale		β‐Values (*P*‐value)
					r. caudate	Not available in this study	−1.536 (0.009)
Ceravolo et al^27^ PD	44	HAMA	^123^I‐FP‐CIT SPECT DAT BR	Partial correlations SPSS	HAMA	Not available in this study	r‐Values (*P*‐value)
					r. caudate		0.311 (<0.05)
					l. caudate		0.323 (<0.05)
					r. putamen		0.356 (<0.05)
					l. putamen		0.309 (<0.05)
Erro et al^26^ aPD naPD	9 25	HADS‐A	^123^I‐FP‐CIT SPECT V3″ value	Comparisons (*t* test), multiple regression SPM, ImageJ	aPD ≥ naPD Lower V3″ in	Not available in this study	*P*‐values
					r. caudate		0.007
					l. caudate		0.001
					l. putamen		0.001
					Correlations HADS‐A		β‐coefficient (*P*‐value)
					r. caudate		−0.47 (0.01)
Moriyama et al^25^ sad_PD nosad_PD	11 21	BSPS	^99m^Tc‐TRODAT‐1 SPECT DAT BR	Comparisons (*t* test), partial correlations SPSS	Correlation BSPS	Not available in this study	r‐Values (*P*‐value)
					l. caudate		0.43 (0.02)
					l. putamen		0.43 (0.02)
					Comparisons: no difference		
Weintraub et al^24^ PD	76	STAI	^99m^Tc‐TRODAT‐1 SPECT DAT BR	Pearson correlations *StatS* package	STAI	Not available in this study	r‐Values (*P*‐value)
					l. ant. Putamen		−0.24 (0.04)
Remy et al^23^ PD	20	STAI	^11^C‐RTI‐32 PET DAT and NAT BR	Correlation with GLM SPM	STAI		*z* Values
					l. Ve. striatum	−18/10/8	−2.72
					l. caudate	−12/14/14	−2.34
					l. LC	−6/−30/−18	−2.70
					r. thalamus	16/−10/16	−2.55
					l. thalamus	−6/−8/12	−2.38
					l. amygdala	−22/0/−10	−2.10
					r. amygdala	−24/4/−14	−2.06
**Metabolic imaging studies**							
Wang et al^31^ aPD naPD HC	13 15 15	HAMA	^18^FDG‐PET Cerebral glucose metabolism	Comparisons (*t* test) SPM	aPD ≥ naPD		*z* Scores
					r. OFC	8/62/18	−3.15
					l. OFC	−4/40/−24	−2.82
					aPD ≥ HC		
					b. dACC	0/50/16	−3.90
					r. OFC	8/62/22	−3.35
					r. dlPFC	8/54/36	−2.94
					r. SMC	60/6/46	−2.83
					l. SMC	−6/8/70	−3.24
					r. SA	8/12/−18	−3.52
					r. putamen	14/14/0	−3.43
					l. caudate	−12/14/6	−2.89
					l. OFC	−10/38/−30	−3.04
					r. vlPFC	24/10/38	−3.11
					naPD > HC		
					r. SMC	50/−2/52	−4.08
					l. SMC	−10/8/66	−3.61
Huang et al^32^ PD HC	26 12	BAI	^18^FDG‐PET Cerebral glucose metabolism	Multiple regression SPM	BAI		*z* Values
					r. caudate	12/10/2	−3.60
					l. caudate	−10/10/4	−3.50

Abbreviations: aPD, PD patients with anxiety; BAI, Beck Anxiety Inventory; BR, binding rate; BSPS, Brief Social Phobia Scale; DAT, dopamine transporter; FDG, fluorodeoxyglucose; fMRI, functional MRI; GLM, generalized linear model; HADS‐A, Hospital Anxiety and Depression Scale, Anxiety Subscale; HAMA, Hamilton Rating Scale for Anxiety; NAT, noradrenaline transporter; naPD, PD patients without anxiety; OFC, orbitofrontal cortex; PD, Parkinson's disease; PD‐L, PD patients with left limbs dominantly affected; PD‐R, PD patients with right limbs dominantly affected; sad, social anxiety disorder; SAS, Self‐Rating Anxiety Scale; SBR, striatal binding ratio; SERT, serotonin transporter; STAI, State‐Trait Anxiety Inventory; SPECT, single‐photon emission computed tomography; SPM, statistical parametric mapping; SPSS, Statistical Package for the Social Sciences; PET, positron emission tomography; V3″ value, specific‐to‐non‐displaceable binding ratio; VBM, voxel‐based morphometry.

Region of interest: ant, anterior; dACC, dorsal anterior cingulate cortex; dlPFC, dorsolateral prefrontal cortex; l, left; LC, locus coeruleus; r, right; SA, subgenual area; SMC, premotor cortex and supplementary motor cortex; Ve, ventral; vlPFC, ventrolateral prefrontal cortex.

### Metabolic Imaging Studies

The 2 metabolic imaging studies included 81 participants, of whom 54 were PD patients and 27 were HCs. In these studies, the cerebral glucose metabolism was analyzed using ^18^FDG‐PET. In 1 study, aPD patients were compared with naPD patients and HCs. The other one correlated the severity of anxiety with metabolic changes. Two different anxiety scales were used: the HAMA and the BAI.

Increased anxiety was associated with reduced cortical FDG metabolism in the OFC, dlPFC, ventrolateral PFC, and the cingulate cortex as well as reduced striatal FDG metabolism (bilateral caudate and right putamen). The results are provided in Table 4.

## Discussion

This review aimed at delineating the brain regions involved in anxiety in PD as identified by studies using 3 types of approaches: anatomical, functional, and metabolic imaging. It revealed that several structures were implied in the pathophysiology of fear. Both anatomical and functional changes occurred in the amygdala, the PFC, the ACC, and the striatum corresponding to both the fear and the limbic cortico‐striato‐thalamocortical circuits. A reduced dopaminergic and noradrenergic BR occurred in the striatum, the amygdala, the thalamus, and the locus coeruleus and a reduced serotoninergic binding in the thalamus.

### The Fear Circuit Is Altered in PD Patients with Anxiety

**FIG. 1:**
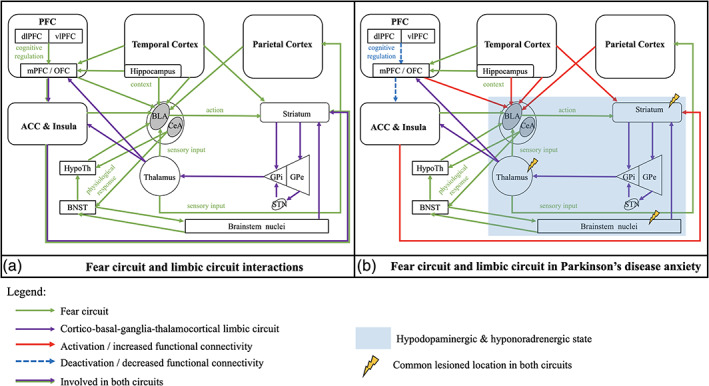
Schematic representation of the fear circuit and cortico‐striato‐thalamocortical limbic circuit in human brain: **(a)** interactions model in normal brain as reported in the literature;^6^, ^8^, ^39^ **(b)** fear and limbic circuit changes in Parkinson's disease anxiety found in this systematic review. The blue area corresponds to the structures with a reduced receptor binding for dopamine and noradrenaline, but no information on structural and functional connectivity changes for the basal ganglia and thalamus has been reported in these studies. Therefore, it does not mean that there is no change. Abbreviations: ACC, anterior cingulate cortex; BLA, basolateral nucleus of amygdala; BNST, bed nucleus of the stria terminalis; CeA, centromedial nucleus of amygdala; GP(e/i), globus pallidus (external/internal); HypoTh, hypothalamus; OFC, orbitofrontal cortex; (m/dl/vl)PFC, (medial/dorsolateral/ventrolateral) prefrontal cortex; STN, subthalamic nucleus. [Color figure can be viewed at wileyonlinelibrary.com]

This review found evidence of anatomical and functional alterations in the fear circuit in PD‐related anxiety. Anatomical and functional changes in the amygdala and a dopaminergic as well as noradrenergic BR reduction were associated with anxiety severity.^16^, ^19^, ^22^, ^23^ The amygdala is the central hub of the fear circuit, commonly separated into 3 nuclei: the centromedial (CeA), the basolateral (BLA), and the superficial nucleus. The BLA is the input nucleus and receives afferent inputs from the PFC, the ACC, the hippocampus, the thalamus, and the brainstem nuclei. It projects to the CeA, the bed nucleus of stria terminalis and the striatum. The CeA is the output nucleus of the amygdala and projects to the brainstem nuclei and the hypothalamus^6^, ^39^ (Fig. 1a). Therefore, an imbalance between the BLA and CeA, with functional dominance of the BLA, could contribute to the occurrence of anxiety symptoms. This review also brought out anatomical and functional changes in the PFC and the ACC. In the fear circuit, theses cortices are postulated to be involved in the cognitive regulation of emotion, whereas the hippocampus is involved in emotional memory and contextual fear reaction.^6^ Other studies also showed that the ventral striatum, the ACC, and the insular cortex could play a crucial role in encoding aversive contextual information and in controlling negative motivation to execute avoidance behavior in response to aversive cues and anticipation of consequence. It was reported that these structures had major inputs from amygdala.^40^, ^41^ Their dysfunction could be associated with impaired voluntary emotion regulation and lower ability to inhibit intrusive negative thoughts. Therefore, it could lead to a disturbance of attentional resources and lower executive performance in anxious PD patients.^42^, ^43^ Functional changes between the hippocampus and amygdala could lead to dysfunction in emotional memory and promote negative thoughts or resurgence of erratic emotional memories. However, dysfunction of the fear circuit is not the only mechanism that can explain the high prevalence of anxiety in PD.

### Changes in Basal Ganglia Circuits Are Involved in PD‐Related Anxiety

The central factor in the neuropathology of PD is dysfunction of the basal ganglia. A hypodopaminergic state of the limbic cortico‐striato‐thalamocortical circuit has been associated with behavioral and psychiatric symptoms in PD, such as anxiety.^8^, ^44^ This circuit connects the ACC, the mPFC, and the brainstem nuclei with the basal ganglia such as the striatum, the pallidum, the subthalamic nucleus (STN), and the thalamus to modulate mood and behavior (Fig. 1a). In this review, functional changes of the striatum were associated with the severity of anxiety. Moreover, anxiety was associated with a reduced dopaminergic, noradrenergic, and serotoninergic BR in the structures involved in the limbic cortico‐striato‐thalamocortical circuit, such as the striatum, the locus coeruleus, and the thalamus. Erro and colleagues^26^ proposed that cognitive and behavioral dysfunctions observed in PD patients might reflect a sequential process of dopamine depletion occurring in the striatum. The relationship between anxiety and hypo‐dopaminergic state in the striatum may be mediated by disruption of the dopaminergic cortico‐striato‐thalamocortical circuit.^26^ In this circuit, the mediodorsal thalamus is an especially important relay between the basal ganglia and the mPFC/ACC, but it also brings sensory input to the BLA and more generally to the fear circuit.^6^, ^8^ The locus coeruleus is the main noradrenergic center in the brain. Remy and colleagues postulated that anxiety in PD could implicate thalamocortical interactions under the control of the noradrenergic innervation originating in the locus coeruleus.^23^ These findings are consistent with the hypothesis of a hypo‐catecholaminergic and hypo‐serotoninergic state of the limbic circuit in PD patients with anxiety. It is thus postulated that the neuropathology of PD itself could affect the pathophysiology of the fear circuit.

### The Neuropathology of PD Increases the Risk of Anxiety

In this review, anxiety in PD was associated with anatomical and functional changes in both the fear circuit and the limbic cortico‐striato‐thalamocortical circuits. We assume that the neuropathology of PD could affect the fear circuit in different ways. First, there is an important overlap between the fear and the limbic circuit. The anatomical separation between these circuits seems artificial. They must be seen as 2 parts of a bigger limbic circuit (Fig. 1b). Dysfunction of the basal ganglia and the hypo‐dopaminergic state due to PD could affect the proper function of the limbic circuit. It could promote an over‐activation of the fear circuit, altering fear processing, as well as an under‐activation of the limbic cortico‐striato‐thalamocortical circuit, altering the cognitive and behavioral long‐term adaptation to fear. Second, dysfunction of these circuits may occur simultaneously or successively in the course of the disease. In this review, anxiety was associated with reduced dopamine, catecholamine, and serotonin in the thalamus and in the locus coeruleus. These structures are both closely connected to the 2 circuits.^6^ On the one hand, the mediodorsal thalamus is directly connected to the BLA and brings sensory input to the fear circuit.^6^, ^39^, ^43^ It is also probably connected to the striatum in the fear circuit, but we did not find any confirmation in literature (Fig. 1a). On the other hand, lesions of brainstem nuclei, such as lesions of the locus coeruleus or the raphe nucleus, occur early in the course of PD^45^, ^46^ and could promote dysfunction of both the cortico‐striato‐thalamocortical circuit and the fear circuit, in parallel or successively. The early impairment of these nuclei could therefore promote anxiety symptoms. It could explain the high prevalence of anxiety and its associated symptoms in PD. Finally, other structures, such as the ventral tegmental area (VTA), the STN, the periaqueductal gray matter, the raphe nuclei, or the parabrachial nuclei, have been identified to be involved in fear and anxiety disorders but have not been studied in PD.^47^ The alterations in limbic circuits in the included studies could also indirectly reflect neuropathological dysfunction of these structures due to the pathology of PD.

### Anxiety, Depression, and Apathy: A “Non‐Motor Triad”

In addition to studies focusing on the imaging of anxiety, studies addressing the border area of anxiety, depression, and apathy may shed light on the neurocircuitry of anxiety. Although not the focus of our search, depression and apathy are commonly associated with anxiety. Some authors suggested that these 3 neuropsychiatric manifestations would constitute a behavioral “non‐motor triad” in PD.^48^ On the one hand, several studies demonstrated that dysfunction of the cortico‐striato‐thalamocortical limbic circuit (OFC, ACC, and limbic part of basal ganglia) is implied in the pathophysiology of apathy, depression, and anxiety. These suggest that a more widespread meso‐cortico‐limbic dopaminergic denervation (OFC, dlPFC, cingulate cortices, left ventral striatum, and right amygdala) is involved in the pathogenesis of apathy and depression.^49^ Moreover, another study stressed the importance of degeneration of serotonergic structures within the limbic system in this “non‐motor triad,” which is already present at the beginning of the disease. The severity of anxiety in apathetic PD patients was linked to a serotonergic disruption within the bilateral ACC, without a prominent role of dopaminergic degeneration.^48^ In our review, one study also showed that the severity of depression, apathy, and anxiety was associated with a loss of dopamine and noradrenaline innervation in the locus coeruleus and the limbic system (ACC, thalamus, amygdala, and ventral striatum).^23^ In another systematic review, the authors confirmed that not only mesolimbic dopaminergic but also mesolimbic serotonergic and noradrenergic lesions play a major role in the mechanisms of these 3 psychiatric symptoms.^9^ On the other hand, several studies showed differences in the underlying mechanisms of depression, apathy, and anxiety. In neurotransmitter imaging studies, these 3 symptoms were associated with a reduced dopaminergic innervation in the striatum, notably the ventral striatum, but several studies showed a specific reduction in the caudate nucleus in anxious PD patients.^23^, ^26^, ^50^ Zhang and colleagues reported a positive correlation between the FC of the left parahippocampal gyrus and the severity of depressive symptoms in PD, whereas the severity of anxiety was positively correlated to the FC between the parahippocampal gyrus and the left amygdala. The functional networks associated with depression and anxiety were also different.^19^, ^22^ Recently, a study using VBM and DTI showed that de novo apathetic PD patients (with or without depression) had microstructural alterations in the medial cortico‐striatal limbic system (striatum, ACC, medial frontal cortex, thalamus, and midbrain). There was no microstructural alteration correlated with symptoms of anxiety.^46^ These studies point out that considering the pathophysiology of anxiety independently of depression and apathy is difficult but that it might have distinct underlying mechanisms. They also highlight the fact that further appropriate studies are needed to decipher these mechanisms.

### Strengths and Limitations

In our review, we strictly followed the PRISMA guidelines for systematic reviews. We did not include the terms “electroencephalography” or “magnetoencephalography” in our search strategy, because this was not considered within the scope of our review. In a post hoc exploratory search, no study used these methods to specifically explore the pathophysiology of anxiety in PD. However, such studies could usefully extend the understanding of the pathophysiology of anxiety in PD.

Anxiety is usually not an isolated symptom. It is often associated with depression, apathy, and/or cognitive decline. It is thus difficult to determine the pathophysiology of anxiety independently of these other neuropsychiatric symptoms (see the section “Anxiety, Depression, and Apathy: A ‘Non‐Motor Triad’ ”). The mean cognitive scores (Mini‐Mental State Examination or Montreal Cognitive Assessment) of the patients in the included studies are provided in Table 1 and show no cognitive decline in our sample. However, there were limitations related to the included studies. All studies were cross‐sectional, which implies that it was not possible to conclude about temporal or causal relations. Moreover, there may be alterations in other structures than those we focused on, such as the VTA and STN. Further studies are needed to identify the involvement of the latter and other structures in PD‐related anxiety. Other limitations of the included studies were inclusion of patients with subclinical anxiety symptoms, the use of nonvalidated clinical rating scales for anxiety, the lack of separation of different anxiety diagnoses, and the lack of correction for covariables. Finally, the lack of a HC group in some of the included studies is also a limitation.

### Conclusion

In this review, anxiety symptoms were associated with alterations of the limbic cortico‐striato‐thalamocortical circuit and the fear circuit. In PD, dysfunction of basal ganglia and brainstem nuclei could lead to alteration in both circuits explaining the high prevalence of anxiety in Parkinson's disease and the motor, behavioral, and cognitive symptoms associated.^3^ Further studies are needed to better understand the pathophysiology of this symptom.

## Author Roles

(1) Research project: A. Conception, B. Organization, C. Execution; (2) Review protocol: A. Design, B. Execution, C. Review and critique; (3) Manuscript: A. Writing of the first draft, B. Review and critique.

G.C.: 1A, 1B, 1C, 2A, 2B, 3A

M.G.: 1C, 2B, 3B

J.J.A.d.J.: 2C, 3C

P.A.M.H.: 2C, 3C

W.H.B.: 2C, 3C

K.D.: 2C, 3C

A.F.G.L.: 1A, 1B, 2A, 2C, 3C

## Supporting information


**APPENDIX S1.** Supporting Information
