# Supplementary material for: Neuroimaging of Anxiety in Parkinson's Disease: A Systematic Review
Source: Mov Disord. 2020 Dec 2;36(2):327–39. doi: 10.1002/mds.28404 (PMC7984351; doi:10.1002/mds.28404)
Supplement: Supplementary file 1 — APPENDIX S1. Supporting Information [file MDS-36-327-s001.docx]

**Neuroimaging of anxiety in Parkinson’s disease: a systematic review**

Guillaume Carey (MD)^1,2^, Meltem Görmezoğlu (MD)^3,5^ , Joost J.A. de Jong (PhD)^1,4^, Paul A.M. Hofman (MD, PhD)^1,4^, Walter H. Backes (PhD)^1,4^, Kathy Dujardin (PhD)^2^, Albert F.G. Leentjens (MD, PhD)^1,3^

^1^School for Mental Health and Neurosciences (MHeNS), Maastricht University, Maastricht, The Netherlands;

^2^ Univ. Lille, Inserm, CHU Lille, Lille Neurosciences and Cognition, Lille, France;

^3^ Department of Psychiatry, Maastricht University Medical Center, Maastricht, the Netherlands;

^4^Department of Radiology and Nuclear Medicine, Maastricht University Medical Center, Maastricht, The Netherlands;

^5^Ondokuz Mayis University Hospital, Department of Psychiatry, Ondokuz Mayıs University, Samsun, Turkey.

**Correspondence**:

Guillaume Carey (e-mail: [g.carey@maastrichtuniversity.nl](mailto:g.carey@maastrichtuniversity.nl))

CHU de Lille, Hôpital Roger Salengro,

Avenue du Professeur Emile Laine, Service de Neurologie A, 59037, Lille, France

**Short title**: Imaging anxiety in PD

**Keywords:** Parkinson’s disease; anxiety; imaging; fear circuit; limbic circuit.

**SUPPLEMENTARY DATA S1**

**Research protocol**

1. Data selection and inclusion criteria

For this systematic review, we included studies (a) with PD-patients that assessed anxiety symptoms, (b) including any method of neuroimaging such as structural or functional MRI or metabolic imaging (PET, SPECT), (c) categorically comparing PD-patients with and without anxiety on imaging parameters, or correlating imaging parameters with severity of anxiety as a continuous variable (d) whose outcome parameters were either gray-matter volume (GMV), the blood-oxygen-level-dependent (BOLD) signal, or the binding-rate (BR) values. Any study design was eligible for inclusion. Also, both studies performing whole brain seed-based analyzes or region of interest (ROI) analyzes were included. The exclusion criteria were: (a) review articles reporting no original data or preclinical studies, (b) studies focusing on other diseases than PD or on anxiety in general, (c) studies without assessment of anxiety, (d) studies without neuroimaging.

Eligibility was determined independently by two researchers (G.C. and M.G.). Firstly, duplicates were removed from the research results and followed by screening of the title and abstract. Discrepancies were resolved in a consensus meeting and a third specialist (A.L.) was consulted if no consensus was reached.

**SUPPLEMENTARY DATA S2**

**Quality assessment procedure**

Two authors (G.C. and M.G.) extracted data. The following information was extracted from each study: the first author, year of publication, journal name, sample size, method of neuroimaging, the anxiety scale used, the statistical methods, the imaging data pre-processing and processing methods, patient characteristics, including age, gender, duration of PD, education, medication and cognitive function. Peak coordinates and effect size measures of the regions with a significant difference in imaging data were also collected if possible. Some publications did not report peak coordinates because of the study design.

To assess risk of bias in individual studies we used nine quality criteria, following the approach of Wolters et al. [1]: (a) patient demographics; (b) imaging procedure; (c) spatial normalization method; (d) determination of ROIs; (e) reproducibility of the analyzes; (f) statistical tests used to substantiate the results; (g) correction for the multiple testing problem; (h) figures and tables; (i) quality control measures (Table S2.1). Studies could score 0, 0.5 or 1 point for each item. An overall score of ≥7.5 was considered as good quality, 4–7.5 as moderate quality, and ≤4 as poor quality [1]. The quality assessment results are detailed on Table S2.2 and Figure S2.1. It was performed by two researchers (G.C. and M.G.) and discrepancies were discussed until consensus was reached. If no consensus could be reached, a third specialist was consulted (A.L.).

The risk of bias across studies could not be assessed formally because the number of included studies was small for each imaging type and/or because the study design was very heterogeneous (comparisons or correlation, different anxiety scale, different metabolic imaging). Moreover, due to this heterogeneity, it was not possible to perform a meta-analysis as we intended initially. Instead, we decided to present a systematic review including peak coordinates and effect size measures.

| a. Did they give a full description of the study participants? |
| --- |
| b. Did they give a full description of the imaging type and acquisition? |
| c. Did they specify the spatial normalization procedure, including the atlas or template which is used to match the images to? |
| d. Did they specify how the regions of interest were determined? |
| e. Did they provide enough detail to reproduce the analyzes? |
| f. Are all the empirical claims supported by a specific statistical test? |
| g. Did they describe and account for the multiple testing problem? |
| h. Do the figures and tables stand on their own? |
| i. Are the quality control measures documented? |

**Table S2.1. Nine quality assessment criteria according to A.F. Wolters et al, 2018** [1]

| Study | a | b | c | d | e | f | g | h | i | Total |  |
| --- | --- | --- | --- | --- | --- | --- | --- | --- | --- | --- | --- |
| Anatomical |  |  |  |  |  |  |  |  |  |  |  |
| Oosterwijk et al., 2018 | 1 | 1 | 1 | 1 | 1 | 1 | 1 | 1 | 1 | **9** |  |
| Ma et al., 2018 | 1 | 1 | 1 | 1 | 1 | 1 | 0.5 | 1 | 0.5 | **8** |  |
| Vriend et al., 2016 | 1 | 1 | 1 | 1 | 1 | 1 | 1 | 1 | 1 | **9** |  |
| Wee et al., 2016 | 1 | 1 | 1 | 1 | 1 | 1 | 1 | 1 | 0 | **8** |  |
| Functional |  |  |  |  |  |  |  |  |  |  |  |
| Zhang et al., 2019 | 1 | 1 | 1 | 1 | 1 | 1 | 1 | 1 | 0 | **8** |  |
| Wang et al., 2018 | 1 | 1 | 1 | 0 | 1 | 1 | 1 | 1 | 0 | **7** |  |
| Dan et al., 2017 | 1 | 1 | 1 | 1 | 1 | 1 | 1 | 0.5 | 0 | **7.5** |  |
| Wang et al., 2017 | 1 | 1 | 1 | 1 | 1 | 1 | 1 | 1 | 0 | **8** |  |
| Metabolic |  |  |  |  |  |  |  |  |  |  |  |
| Bayram et al., 2019 | 1 | 1 | 1 | 0.5 | 1 | 1 | 1 | 1 | 0 | **7.5** |  |
| Joling et al., 2018 | 1 | 1 | 1 | 1 | 1 | 1 | 1 | 1 | 0 | **8** |  |
| Picillo et al., 2017 | 1 | 1 | 1 | 0.5 | 1 | 1 | 0.5 | 1 | 0 | **7** |  |
| Wang et al., 2017 | 1 | 1 | 1 | 0.5 | 1 | 1 | 0 | 1 | 0 | **6.5** |  |
| Ceravolo et al., 2013 | 1 | 1 | 0.5 | 1 | 1 | 1 | 0.5 | 1 | 0 | **7** |  |
| Huang et al., 2013 | 1 | 1 | 1 | 0.5 | 1 | 1 | 1 | 1 | 0 | **7.5** |  |
| Erro et al., 2012 | 1 | 1 | 1 | 1 | 1 | 1 | 0.5 | 1 | 0 | **7.5** |  |
| Moriyama et al, 2011 | 1 | 1 | 0.5 | 1 | 1 | 1 | 0 | 1 | 0 | **6.5** |  |
| Weintraub et al., 2005 | 0.5 | 1 | 0.5 | 0.5 | 1 | 1 | 0.5 | 1 | 0 | **6** |  |
| Remy et al., 2005 | 1 | 1 | 1 | 1 | 1 | 1 | 1 | 1 | 0 | **8** |  |

**Table S1.2. Quality assessment of the studies as good (green), fair (yellow) or poor (red) quality.**

0 = poor criteria; 0.5 = moderate criteria; 1 = good criteria; QC = quality control.


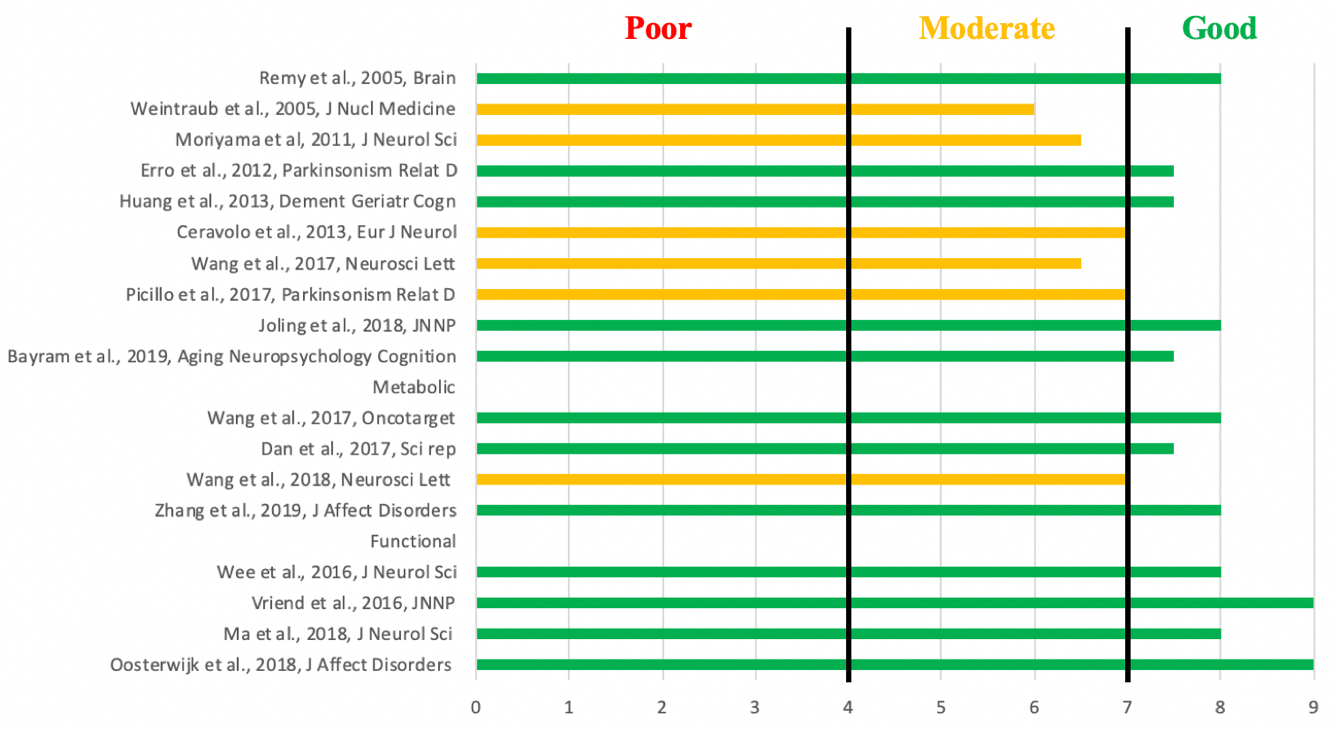


**Figure S1.1. Quality assessment according to A.F. Wolters et al, 2018** [1] **nine criteria.**

An overall score of ≥7.5 was considered as good (green), 4-7.5 as fair (orange) and ≤4 as poor (red) quality.

**SUPPLEMENTARY RESULTS S3:**

**Flow chart of the review**

**
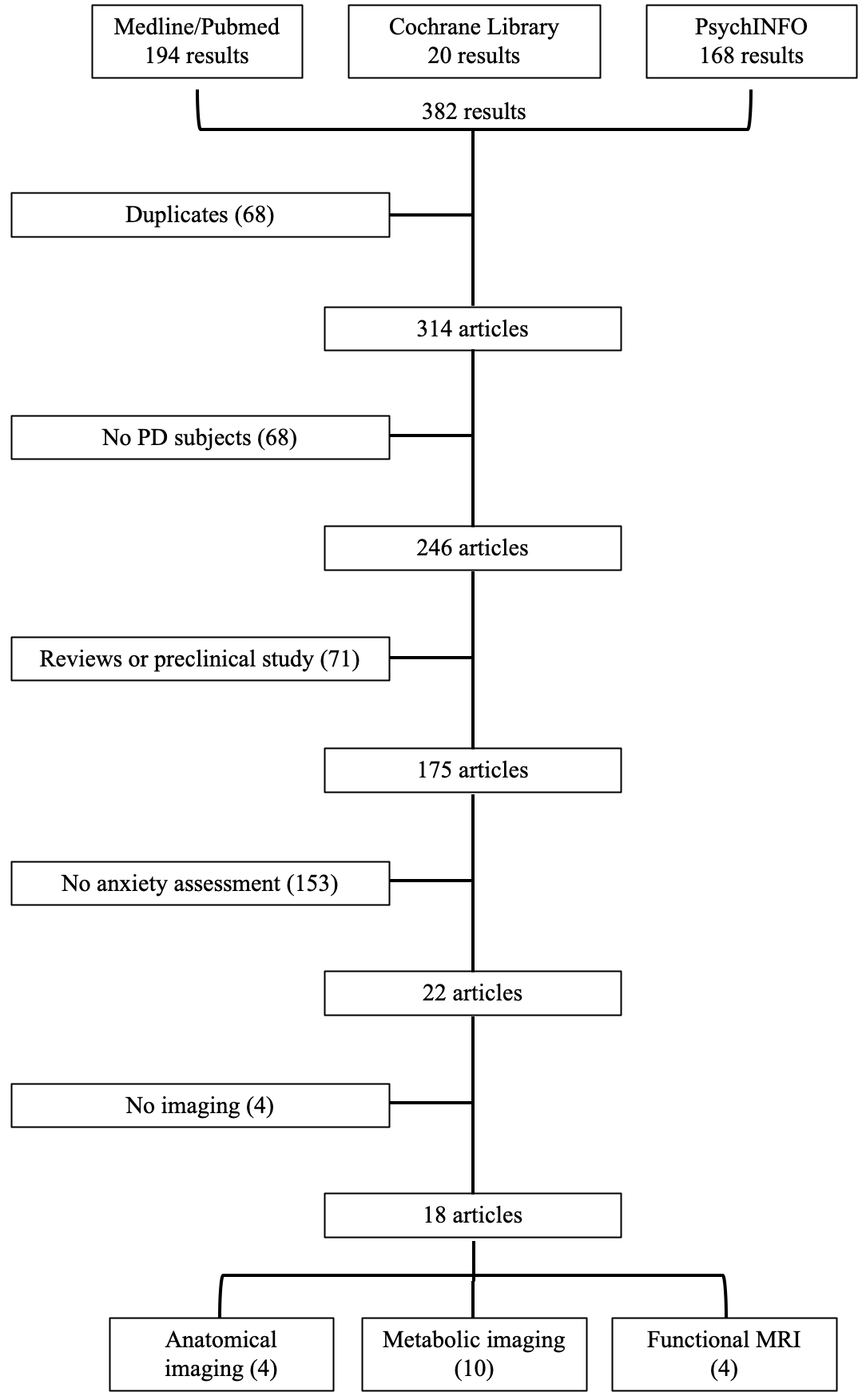
**

**Figure S2.1. Flow chart and research procedure of the systematic review.**

*PD = Parkinson’s disease.*

**References**

1. Wolters AF, van de Weijer S, Leentjens A, Duits AA, Jacobs H, Kuijf ML (2018) Resting-state fMRI in Parkinson’s disease patients with cognitive impairment: A meta-analysis. Parkinsonism Relat D. https://doi.org/10.1016/j.parkreldis.2018.12.016
